# Reply to the letter from Finsterer and Stöllberger “Exhaustion or fatigability may not only be cardiac but also myopathic”

**DOI:** 10.1007/s12471-015-0684-7

**Published:** 2015-03-27

**Authors:** A. Yaksh, N.M.S. de Groot

**Affiliations:** Department of Cardiology, Erasmus Medical Center, Thorax Center, ’s Gravendijkwal 230, 3015 CE Rotterdam, The Netherlands

We are pleased to have received comments on our recently published manuscript ‘Unexpected finding in an adult with ventricular fibrillation and an accessory pathway: noncompaction cardiomyopathy’ [1].

Our patient is indeed not the first patient to be described with the combination of isolated left ventricular noncompaction cardiomyopathy (INVM) and Wolff–Parkinson–White syndrome; however, she is the first presenting an *out-of-hospital cardiac arrest* due to ventricular fibrillation. In the other reports, patients presented with either noncompaction cardiomyopathy or were diagnosed with this disorder by family screening. Feldt et al. [2] described a patient with a spongy trabecular pattern of the inner layer of both ventricles (though more pronounced in the right ventricle), but she also had dextrocardia, subpulmonary stenosis, and ventricular septum defect. In our introduction, we refer to the article of Engberding et al. [3] as the first to describe the presence of an enlarged left ventricle, thickening of the left ventricular wall, and huge sinusoids but a normal right ventricle. Hence, the only anomaly was an ‘isolated noncompaction of the left ventricle’. We agree that Feldt et al. were the first to describe a noncompaction cardiomyopathy (not isolated).

A wide variety of ECG abnormalities and clinical findings can indeed be found in INVM patients, but there were only a few present in our case.

It was the initial assumption on admission that ventricular fibrillation was induced by atrial fibrillation conducting rapidly across the accessory pathway. However, as we found another explanation and there was no documentation of atrial fibrillation during admission, we did not give oral anticoagulation. The patient is a hockey player without any restraints and it is therefore unlikely that she also had neuromuscular disorders. She was examined by a neurologist on admission, who did not find any abnormalities. We therefore think that further neurological evaluation is not indicated. So far, tachyarrhythmias have not been documented and genetic abnormalities have not been found.
